# Medicinal plants used for management of malaria among the Luhya community of Kakamega East sub-County, Kenya

**DOI:** 10.1016/j.jep.2016.08.050

**Published:** 2016-12-24

**Authors:** Nillian Mukungu, Kennedy Abuga, Faith Okalebo, Raphael Ingwela, Julius Mwangi

**Affiliations:** a Department of Pharmacology and Pharmacognosy, University of Nairobi, P.O. Box 19676-00202, Nairobi, Kenya; b Department of Pharmaceutical Chemistry, University of Nairobi, P.O. Box 19676-00202, Nairobi, Kenya

**Keywords:** AIDS, Acquired Immune Deficiency Syndrome, APHRC, African Population and Health Research Center, CARTA, Consortium for Advanced Research Training in Africa, DelPHE, Development Partnerships in Higher Education, DfID, Department for International Development, HIV, Human Immunodeficiency Virus, IC_50_, Half Maximal Inhibitory Coefficient, KIPPRA, Kenya Institute for Public Policy Research and Analysis, KNBS, Kenya National Bureau of Statistics, NCAPD, National Coordinating Agency for Population and Development, PMI, President's Malaria Initiative, UK, United Kingdom, USAID, United States Agency for International Development, WHO, World Health Organization, Ethnopharmacology, Malaria, Medicinal plants, Kakamega East, Luhya

## Abstract

**Background:**

Malaria remains a major health problem worldwide especially in sub-Saharan Africa. In Kenya, 80% of the population is at risk of contracting the disease. Pregnant mothers and children under five years are the most affected by this disease. Antimalarial drug resistance poses a major threat in the fight against malaria necessitating continuous search for new antimalarial drugs. Due to inadequate and inaccessible health facilities, majority of people living in rural communities heavily depend on traditional medicine which involves the use of medicinal plants for the management of malaria. Most of these indigenous knowledge is undocumented and risks being lost yet such information could be useful in the search of new antimalarial agents.

**Aim of study:**

An ethnobotanical survey was carried out among the Luhya community of Kakamega East sub-County, a malaria epidemic region, with the aim of documenting the plants used in the management of malaria.

**Materials and methods:**

Semi-structured questionnaires were used to collect information from 21 informants who included traditional medicine practitioners and other caregivers who had experience in use of plants in management of malaria. These were drawn from 4 villages located in Kakamega East sub-county, within Kakamega County based on their differences in topography. Information recorded included plant names, parts used, mode of preparation and administration and the sources of plant materials. A literature search was conducted using PubMed and google scholar to identify the reported traditional uses of these plants and studied antiplasmodial activities.

**Results:**

In this study, 57% of the informants were aged above 50 years and a total of 61% had either no formal education or had only attained primary school education. A total of 42 plant species belonging to 24 families were identified. Most plants used in the management of malaria in this community belonged to Lamiaceae (18%), Leguminosae (9%) and Compositae (9%) plant families. Plants mostly used included *Melia azedarach* L*,* Aloe spp, *Ajuga integrifolia* Buch. Ham, *Vernonia amygdalina* Del., *Rotheca myricoides* (Hochst.) Steane and Mabb, *Fuerstia africana* T.C.E.Fr., *Zanthoxylum gilletii* (De Wild.) P.G.Waterman and *Leucas calostachys* Oliv. *Rumex steudelii* Hochst.ex A. Rich and *Phyllanthus sepialis* Müll. Arg are reported for the first time in the management of malaria. Although *Clerodendrum johnstonii* Oliv. (Jeruto et al., 2011) and *Physalis peruviana* L.(Ramadan et al., 2015) are reported in other studies for management of malaria, no studies have been carried out to demonstrate their antiplasmodial activity.

The plant parts mostly used were the leaves (36%) and stem barks (26%). Majority of these plants were prepared as decoctions by boiling and allowed to cool before administration (66%) while infusions accounted for 28% of the preparations. The literature mined supports the use of these plants for the management of malaria since most of them have demonstrated *in-vitro* and *in-vivo* antiplasmodial activities.

**Conclusion:**

Most of the reported plant species in this study have been investigated for antiplasmodial activity and are in agreement with the ethnomedical use. Two (2) plants are reported for the first time in the management of malaria. There is need for documentation and preservation of the rich ethnomedical knowledge within this community given that most of the practitioners are advanced in age and less educated. There is also the danger of over-exploitation of plant species as most of them are obtained from the wild, mainly Kakamega forest. Therefore, there is need for determining the economically and medicinally important plants in this community and planning for their preservation.

## Graphical abstract

**Figure:**
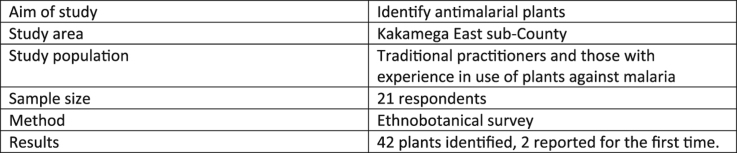

## 1 Introduction

The World Health Organization (WHO) estimates that 3.2 billion people are at risk of malaria infection globally. In the year 2015, a total of 214 million cases and 438,000 deaths due to malaria were reported globally. The burden was highest in WHO-Africa region where 90% of all malaria death occurred (WHO, 2015). In Kenya malaria, still remains a major health problem with 80% of the population at risk of contracting the disease (PMI, 2015). Malaria affects the poor and marginalized populations. In most cases such populations live in rural areas and lack access to adequate healthcare facilities (Yusuf et al., 2010). Therefore, such people largely depend on herbal medicines for the management of malaria and other diseases.

Kakamega county lies within the western highlands malaria epidemic region in Kenya (KNBS and ICF Macro, 2011 and USAID, 2013). This region experiences seasonal malaria outbreaks. The epidemics are favored by the high temperature of above 18 °C during the long rainy seasons which is optimal for breeding of mosquitoes. The poverty level in this county is estimated at 57% (NCAPD, 2005). The high malaria incidences, high poverty levels and the proximity to the tropical forest, Kakamega forest, promotes the use of plants in management of various ailments including malaria.

Most of the African societies have a long history of indigenous healing practices. This knowledge is often passed from generation to generation by word of mouth. The Luhya community in Kakamega has a rich culture of herbalism. The practitioners of herbal medicine are well known within the community and are sought after for their skills in management of diseases. However other members of the community who are not herbal practitioners too have some knowledge on use of plants in management off common diseases and only employ within family context such as mothers in management of children illnesses without consulting the recognized herbalists (Wane, 2011). This knowledge is likely to be lost if not documented. This study therefore sought to document the ethnopharmacological knowledge in the management of malaria in Kakamega County.

## 2 Methods

### 2.1 Study area

**Fig. 1:**
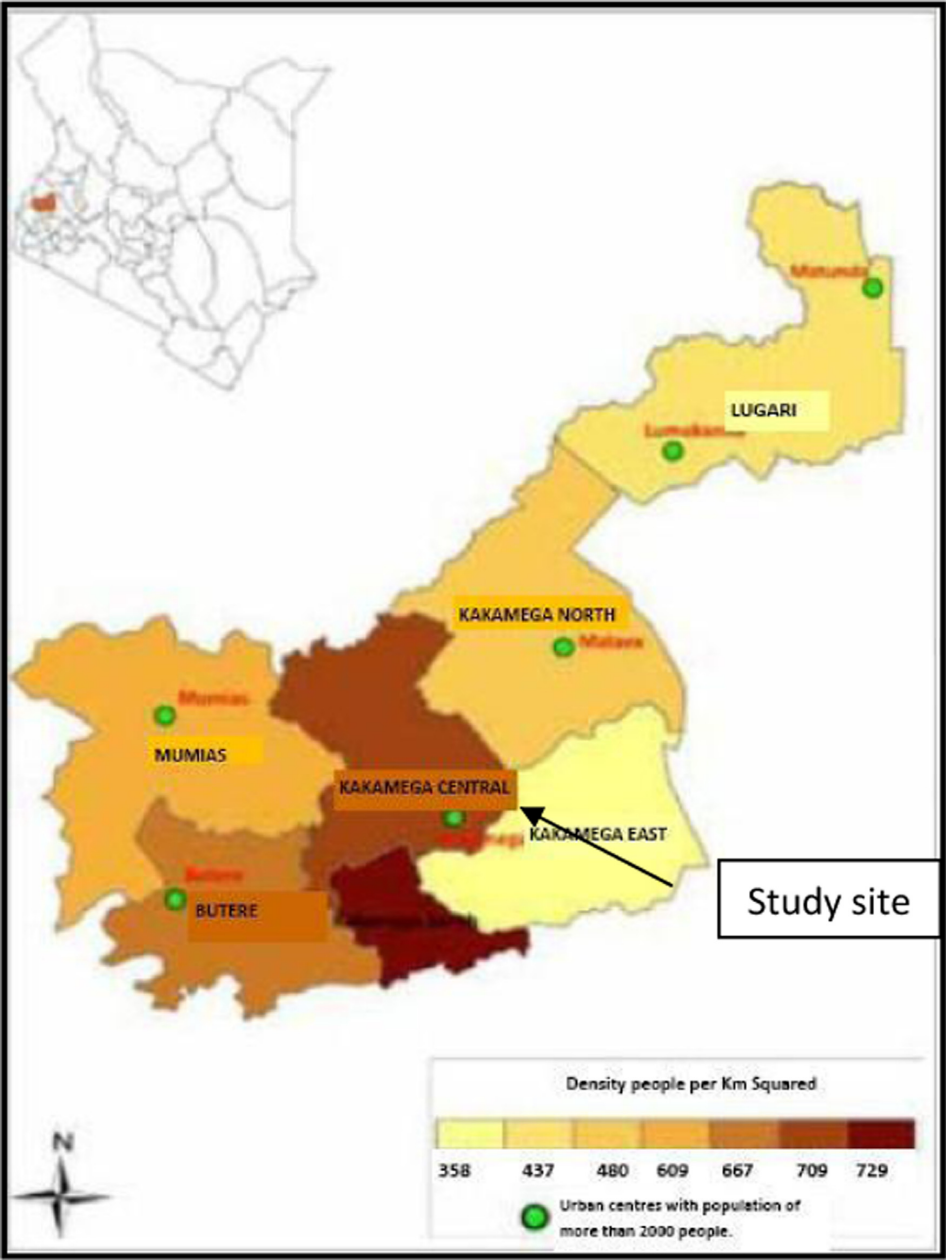
Map of Kakamega county showing study site. (Source: CRA, 2013)

The ethnopharmacological survey was carried out in Kakamega sub-county in Kakamega County. Kakamega County is located in Western Kenya (Fig. 1). The county lies within the longitudes 34° 20′ 35.29″ E- 35° 09′ 27.04″ E and latitudes 0° 05′ 19.12″N- 0° 53′ 53.81″ N. It boarders several other counties, Bungoma to the North, Trans Nzoia to the North East, Uasin-Gishu and Nandi Counties to the East, Vihiga to the South, Siaya to the South West and Busia to the West. According to the 2009 census, the county has a total population of 1,660,651 people with population density of 515 people per km^2^. The poverty level in the county is estimated to be 57% (KIPPRA, 2013).

Kakamega East District, the focus of this study is one of the 6 districts in Kakamega County. The district is mainly a rural set-up with no single urban center. The entire district is served with only dispensaries and health centers (NCAPD, 2005). The nearest high level health facility, Kakamega County hospital, 20 km away.

Kakamega East District is the home to the Kakamega forest, the only tropical rain forest in Kenya. This forest is the main source of herbal medicines for rural communities in the region (Nyunja et al., 2009). The poor health facilities and proximity to the forest are promote the use of herbal medicines in this community.

### 2.2 Ethical approval for the study

Ethical approval for the ethnobotanical survey was obtained from the Kenyatta National Hospital/University of Nairobi Ethics and Research Committee (P186/03/2015). The community gate keepers, who included village elders and church leaders were consulted and subsequently approved the study to be conducted within the local villages. The participants in this study were provided with information on the nature of study, benefits and risks involved. Those who agreed to participate signed a written consent at the beginning of the study.

### 2.3 Ethnobotanical survey

An ethnobotanical survey was carried out between the August and October 2015 in Kakamega East Sub-county of Kakamega County. The region was subdivided into 4 villages based on the differences in topography. Interviews were conducted using semi-structured questionnaires. The study was concluded when no more new information was realized. A total of 21 respondents, both male and female, who utilized antimalarial plants either for self-medication or for treating others were interviewed. A research assistant known to the locals accompanied the researchers during the interviews. Voucher specimens were prepared for all plants collected and deposited at the Department of Botany, University of Nairobi.

## 3 Results and discussion

### 3.1 Socio-economic characteristics of respondents

**Fig. 2:**
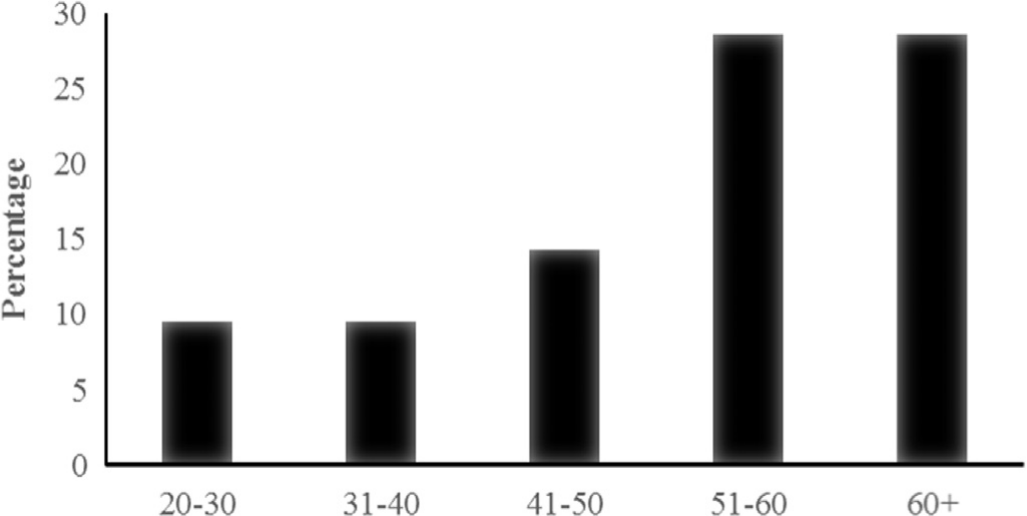
Age Groups of Study Respondents.

The majority (57%) of respondents in this survey were male aged >50 years of age (Fig. 2). The age ranged between 21 and 85 years. Usually the older members of the society have experience in the practice of traditional medicine and pass it on to the younger generation. The younger generation are also not readily accepted by the community as traditional practitioners as they are considered inexperienced (Lambert et al., 2011). In this study, the older practitioners were more recognized by the community than the younger ones. This explained why more than half the respondents were advanced in years.

**Fig. 3:**
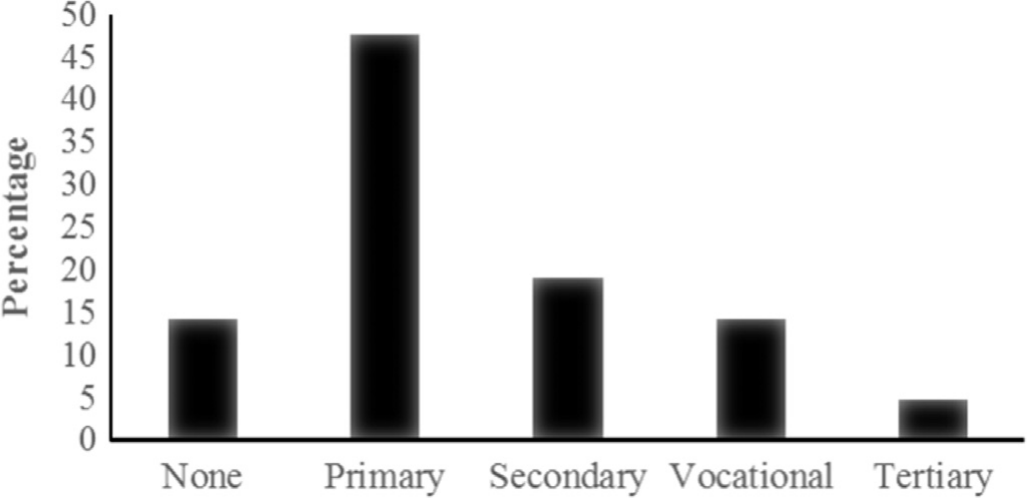
Education Level of Respondents.

Most of the respondents had a primary school level of education (Fig. 3). The practice of traditional medicine has been for a long time been restricted to the less educated since the most educated people view it as ancient form of medicine that is primitive and inappropriate. Most of the practitioners only charge a small fee or no fee at all in managing the common diseases such as malaria since the plants are obtained locally making it not a lucrative business.

### 3.2 Source of Ethnomedical Knowledge

Majority of the respondents (48%) had acquired the knowledge of the practice of traditional medicine from the older members of their families such as parents and grandparents. However, a relatively large proportion (43%) had acquired the knowledge though other means such as apprenticeship under practicing herbalists or by reading books about traditional medicine. Only 1 person had acquired the knowledge through formal training. This is similar to findings in other studies where apprenticeship is the commonest means of learning traditional practices (Lambert et al., 2011). In the recent years, there has been a global increase in demand and acceptability of traditional medicine (Abdullahi, 2011). In view of this, there is increase in commercialization of herbal medicines and more people learning about herbal medicine as a source of income. This may be the reason for the higher number (52%) of people in this study who practice herbalism even though they did not inherit the practice.

### 3.3 Antimalarial plants diversity

Most of the respondents in this study identified fever as the main symptom associated with malaria. Other symptoms mentioned included headache, vomiting, diarrhea and joint pains. They were also aware of severe form of malaria (cerebral malaria). Only one respondent claimed he could treat cerebral malaria. The rest indicated that such cases should be referred to hospital.

**Fig. 4:**
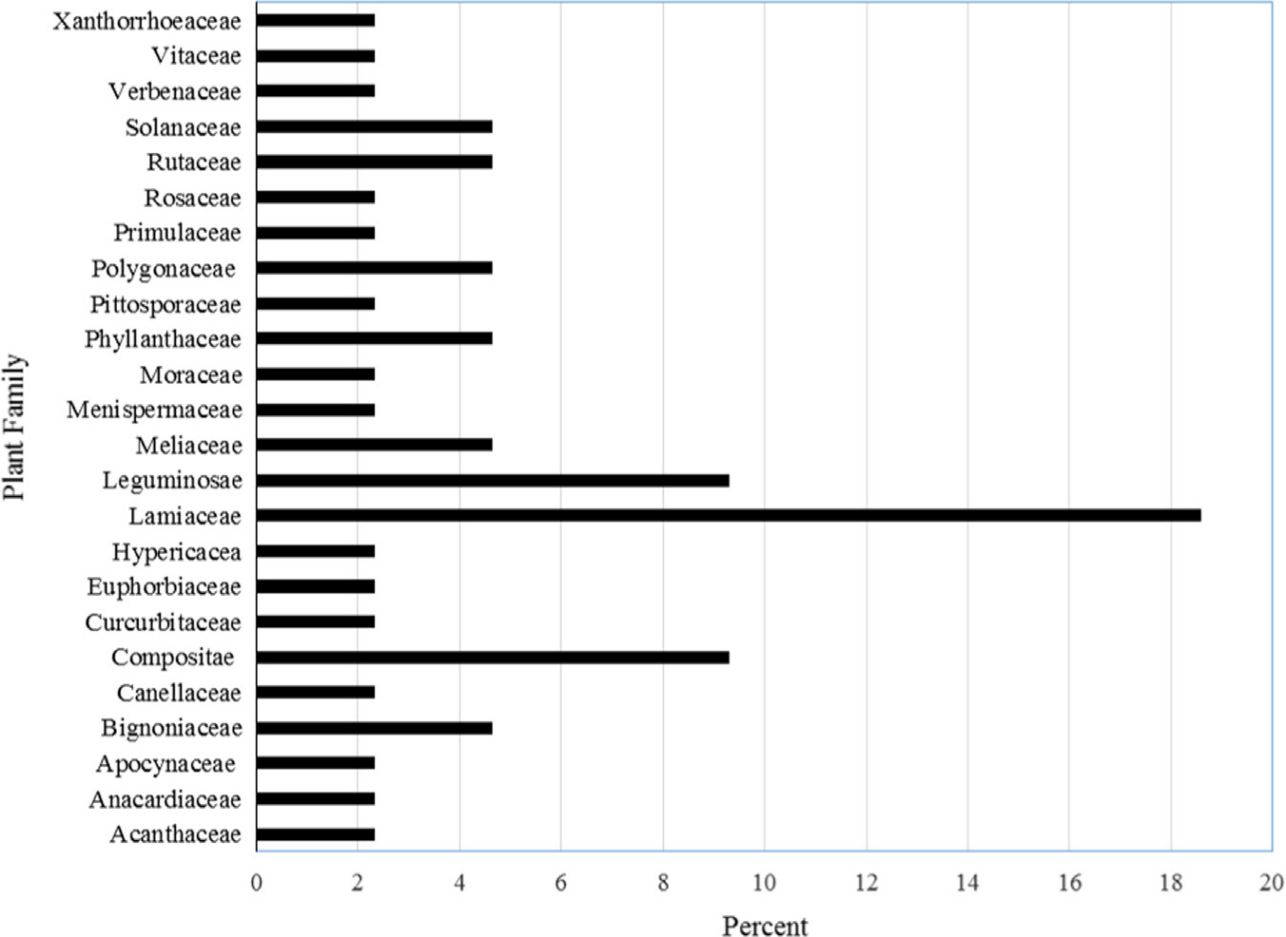
Frequency of use of plant families in management of malaria in Kakamega East sub-County.

A total of 42 plant species belonging to 39 genera within 24 families were identified (Table 1). A large proportion of these plants were from the Lamiaceae (18%), Leguminosae (9%), and Compositae (9%) families (Fig. 4). Most of these plants were shrubs (42%) and trees (27%) followed by climbers (24%) and herbs (7%). Plants mostly cited included *Melia azedarach* L*, Ajuga integrifolia* Buch.-Ham and *Aloe* spp. *Rumex steudelii* Hochst.ex A. Rich and *Phyllanthus sepialis* Müll. Arg are reported for the first time in the management of malaria.

More than 90% of the plants were referred to by their local names. However four of the plants could not be identified by their local names indicating that they may have been introduced into the region. *Justicia betonica* L. was referred to as the dark “*Imbuli yu mutakha*” which refers to *Ajuga integrifolia* Buch.-Ham that is commonly known in this region. The association of this plant with known plant could be due to the use for malaria or due to the bitter taste associated with both plants. However, the 2 plants have very different morphological characteristics. *Cucumis aculeatus* was referred to as *“Khaseveve”* because of having leaves with morphological similarity to a pumpkin plant's leaves (*Liseveve)*.

**Fig. 5:**
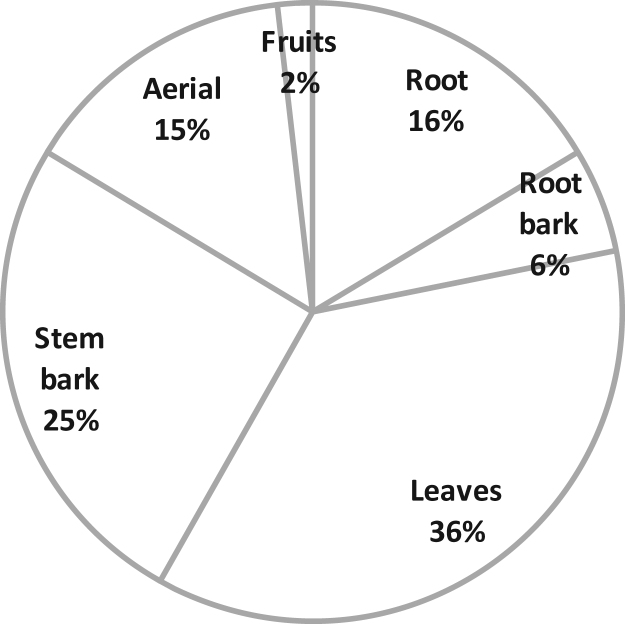
Plant parts used in malaria management.

The part of plant used as medicine plays an important role in sustainability of herbal medicine. The most commonly used plant parts were the leaves (36%) and stem barks (25%) (Fig. 5).

The use of roots and root bark is not sustainable for medicinal plants since the plant is uprooted in most cases to obtain these parts. In this study, roots and root barks plant parts accounted for 22% of all the plants identified. Conservationists warn of over-exploitation of medicinal plants which are valued for their root parts and stem barks (Maroyi, 2013). In this study a total of 47% of the plants were valued for the root parts or stem barks therefore threatened by over-exploitation. Leaves and fruits are the most preferred parts for sustainable plant use since they are the least destructive to the plant and they accounted for 38% in this study.

### 3.4 Preparation of antimalarial medicines

Various methods are used in preparation of herbal medicine among the community. The most common preparation were decoctions (70%), which were made by boiling plant material before use. Other methods included cold maceration (30%), steaming (12%), roasting (8%) to obtain ash or chewing (8%). In most of the cases, the plant material was harvested and prepared just before use. Nevertheless, where the plants are not easily accessible, material was preserved by air-drying under shade and stored for future use.

### 3.5 Sources of plant material

The herbal medicines used for malaria were mainly obtained from the wild (77%) with only 23% cultivated. The cultivation of medicinal plants was mainly done for those plants not easily available in the community, the introduced plants or those that face extinction. In this study, *Justicia betonica* L. an introduced herb, was mainly planted along river beds. *Ajuga integrifolia* Buch.-Ham which almost faces extinction was also planted by the herbalists. Previous studies carried out in Kenya show that most of the herbal products are exclusively obtained from the wild. This strongly indicates the unsustainability of herbal practice in Kenya (McMullin et al., 2012).

### 3.6 Reported traditional uses and antiplasmodial activity

The identified plants in this study have been used in many communities for the management of various ailments including malaria and other febrile illnesses. A total of 38 out of the 42 identified plants have been tested in the laboratory for *in-vivo* and/or *in-vitro* antiplasmodial activities as summarized in Table 2. In vitro antiplasmodial activity is classified as high (IC_50_<5 μg/ml), promising (5<IC_50_<15 μg/ml), moderate (15<IC_50_<50 μg/ml) and inactive (IC_50_>50 μg/ml) (Lekana-Douki et al., 2011). Based on this criteria, 14 of the plants from our study are classified as possessing high antiplasmodial activity with *Albizia gummifera* (J.F.Gmel.) C.A.Sm.*, Leucas calostachys* Oliv., *Tithonia diversifolia* and *Harungana madagascariensis* Lam. ex Poir. having the highest antiplasmodial activity of <1 μg/ml. Although *Tithonia diversifolia* (Hemsl.) A. Gray has promising antiplasmodial activity, study by Elufioye et al. (2009) showed that it is toxic to the liver and kidney therefore limiting its widespread use in the management of malaria.

Although *Clerodendrum johnstonii* Oliv. (Jeruto et al., 2011) and *Physalis peruviana* L. (Ramadan et al., 2015) are reported in other studies for management of malaria, no studies have been carried out to demonstrate their antiplasmodial activity. Less than half of these plants have antiplasmodial compounds isolated as shown in Table 2.

Several ethnobotanical studies to identify antimalarial plants have been carried out in Kenya. These studies identify a variety of plants used by different ethnic communities or regions in Kenya. They include the Nandi (Jeruto et al., 2011), Digo in Kwale (Muthaura et al., 2007), Msambweni (Nguta et al., 2010), Kisumu (Orwa et al., 2007), Central Kenya communities (Njoroge and Bussmann, 2006) and Kilifi (Gathirwa et al., 2011). This is the first study to document antimalarial plants used among the Luhya community in Kenya.

The different Kenyan communities utilize plants within the local communities in the management of malaria. The differences in geographical and climatic conditions which determine the flora available in a given region. However some plants have a wider distribution and therefore most likely used by most communities. For instance, *Aloe* species and *Melia azedarach* L. are plants utilized in all these studied communities. Very few similarities were observed in the utilization of plants among the Luhya community and the Coast Kenya regions communities (Msambweni and Kilifi). For instance, only 3 plants were used in by both the Luhya and Kilifi communities.

Several similarities were observed in the antimalarial plants used in Central Kenya, Nandi and the Luhya community. For instance, 30% of the plants used by the Luhya community were also utilized by the Nandi community. The Nandi and Luhya communities boarder each other. The similarities in the use of the plants could be either due to similarities in vegetation as a result or similar climatic conditions. It could also result from the interchange of knowledge within communities. It is therefore important to consider factors such as ecology, culture and even religious context in the protection of indigenous knowledge and not just a matter of ethnic group. To address this, the concept of ‘Collective Biocultural Heritage’ (BCH) was developed to consider cases where different communities share traditional knowledge within given shared territories and resources. This concept has been utilized in Peru where Inter-Community Agreement for equitable benefit sharing between 6 communities (Swiderska, 2007).

## 4 Conclusion

This study provides a documentation of plants used in the management of malaria in the Luhya community of Kakamega East sub-county. Most of the plants cited in this study have been reported elsewhere for management of malaria and have demonstrated antiplasmodial activities. This indicative of the rich nature of ethnomedical knowledge in this community. *Rumex steudelii* Hochst.ex A. Rich and *Phyllanthus sepialis* Müll. Arg are reported for the first time in the management of malaria. There is therefore need to preserve the ethnomedicine knowledge from this community given that most of the practitioners of traditional medicine are older generation with less education.

Conservation methods need to be put in place to secure the future of traditional medicine practice in this community. The current status of harvesting from the wild and use of roots and barks should be done in a sustainable manner. Members of the community should be educated on sustainable harvesting and cultivation of medicinal plants.

## Funding

This research was supported by the Consortium for Advanced Research Training in Africa (CARTA). CARTA is jointly led by the African Population and Health Research Center (APHRC) and the University of the Witwatersrand and funded by the Wellcome Trust (UK) (Grant No: 087547/Z/08/Z), the Department for International Development (DfID) under the Development Partnerships in Higher Education (DelPHE), the Carnegie Corporation of New York (Grant No: B 8606), the Ford Foundation (Grant No: 1100-0399), Google. Org (Grant No: 191994), Sida (Grant No: 54100029), MacArthur Foundation (Grant No: 10-95915-000-INP) and British Council.

## Figures and Tables

**Table 1:** Plants used in the management of malaria among the Luhya community, Kakamega East sub-County.

**Voucher number**	**Family**	**Plant name**	**Local name**	**Growth form**	**Plant part used**	**Frequency of citation (%)**	**Mode of preparation**
NMA2015/01	Acanthaceae	*Justicia betonica* L.	–	Herb	Aerial	14.3	Pound, add cold water/Boil
NMA2015/02	Anacardiaceae	*Searsia natalensis* (Bernh.ex C. Krauss)	*Busangula*	Herb	Leaves, Stem Bark	4.8	Boil in water
NMA2015/03	Apocynaceae	*Carissa edulis* L.	*Shikata/Achoka*	Shrub	Root Bark	4.8	Boil in water/Inhale steam
NMA2015/04	Compositae	*Acmella caulirhiza* Del.	*Shituti*	Herb	Aerial Part	9.5	Pound, add cold water
NMA2015/05	Compositae	*Microglossa pyrifolia* (Lam.) Kuntze	*Ing’oi*	Shrub	Root, Leaves	9.5	Boil in water
NMA2015/06	Compositae	*Tithonia diversifolia* (Hemsl.) A. Gray	*Masambu malulu/libinzo*	Shrub	Leaves	9.5	Pound, add cold water
NMA2015/07	Compositae	*Vernonia amygdalina* Del.	*Musulilitsa*	Shrub	Leaves	19.0	Pound in cold water/ Boil
NMA2015/08	Bignoniaceae	*Markhamia lutea* (Benth.) K.Schum.	*Lusiola*	Tree	Stem Bark	9.5	Boil in water
NMA2015/09	Bignoniaceae	*Spathodea campanulata* P.Beauv.	*Mutsulio*	Tree	Stem Bark	14.3	Boil without crushing
NMA2015/10	Canellaceae	*Warbugia ugandensis* Sprague	*Apachi*	Tree	Leaves, Stem Bark	14.3	Boil in water
NMA2015/11	Curcurbitaceae	*Cucumis aculeatus* Cogn	–	Climber	Leaves	4.8	Pound, add cold water
NMA2015/12	Euphorbiaceae	*Crotom macrostachuys* Hochst. ex Del.	*Musutsu*	Tree	Stem Bark	4.8	Boil in water
NMA2015/13	*Leguminosae*	*Albizia gummifera* (J.F.Gmel.) C.A.Sm.	*Musenzeli*	Tree	Stem Bark	9.5	Boil without crushing
NMA2015/14	*Leguminosae*	*Erythrina abyssinica* DC.	*Murembe*	Tree	Stem Bark	4.8	Boil in water
NMA2015/15	*Leguminosae*	*Senna didmobotrya* (Fresen.) H.S.Irwin and Barneby	*Lubinu*	Shrub	Leaves	4.8	Boil in water
NMA2015/16	*Leguminosae*	*Senna occidentalis* (L.) Link	*Imbindi*	Shrub	Root	4.8	Pound, add cold water
NMA2015/17	Hypericaceae	*Harungana madagascariensis* Lam. ex Poir.	*Musila*	Tree	Stem Bark	4.8	Boil in water
NMA2015/18	Lamiaceae	*Ajuga integrifolia* Buch.-Ham.	*Imbuli yu mtakha*	Herb	Aerial	23.8	Pound, add cold water
NMA2015/19	Lamiaceae	*Clerodendrum johnstonii Oliv.*	*Shiteng’oteng’o*	Shrub	Leaves	4.8	Pounded in cold water/Boil
NMA2015/20	Lamiaceae	*Rotheca myricoides* (Hochst.) Steane and Mabb.	*Shisilangokho*	Shrub	Rootbark, Leaves	19.0	Boil in water/Roast
NMA2015/21	Lamiaceae	*Fuerstia africana* T.C.E.Fr.	*Muvesemu*	Herb	Aerial	19.0	Boiled or roasted
NMA2015/22	Lamiaceae	*Leucas calostachys* Oliv.	*Lumetsani*	Herb	Aerial	19.0	Pound, add cold water/Boil/Steam
NMA2015/23	Lamiaceae	*Ocimun kilimandscharicum* Gürke	*M’monyi*	Herb	Aerial	4.8	Inhale steam
NMA2015/24	Lamiaceae	*Plectranthus barbatus* Andrews	*Shilokha*	Shrub	Leaves	9.5	Chew bud/boil in water
NMA2015/25	Meliaceae	*Mellia azedarach* L	*Muarubaini*	Tree	Leaves, Stem Bark	47.6	Boil in water
NMA2015/26	Meliaceae	*Trichilia emetica* Vahl	*Munyama*	Tree	Stem Bark	4.8	Boil in water
NMA2015/27	Menispermaceae	*Cissampelos mucronata* A.Rich.	*Mukoye*	Climber	Root	4.8	Chewing
NMA2015/28	Moraceae	*Ficus thonningii* Blume	*Mutoto*	Tree	Stem Bark	9.5	Boil in water
NMA2015/29	Primulaceae	*Maesa lanceolata* Forssk.	*Mushevesheve*	Tree	Root Bark, Stem Bark	4.8	Boil in water
NMA2015/30	Phyllanthaceae	*Flueggea virosa* (Roxb. ex Willd.) Royle	–	Shrub	Aerial	4.8	Boil in water
NMA2015/31	Phyllanthaceae	*Phyllanthus sepialis* Müll. Arg.	–	Shrub	Leaves	4.8	Boil in water
NMA2015/32	Pittosporaceae	*Pittosporumviridiflorum* Sims	*M’monyo/Mkungune*	Shrub	Leaves, Stem Bark	4.8	Boil in water
NMA2015/33	Polygonaceae	*Rumex abyssinicus* Jacq.	*Shikachi*	Herb	Leaves	9.5	Pound, add cold water
NMA2015/34	Polygonaceae	*Rumex steudelii* Hochst.ex A. Rich	*Alukhava*	Herb	Root	9.5	Pound, add cold water
NMA2015/35	Rosaceae	*Rubus pinnatus* Wild.	*Butunduli*	Shrub	Leaves, Fruits	4.8	Pounded in cold water/chew
NMA2015/36	Rutaceae	*Clausena anisata* (Willd.) Hook.f. ex Benth.	*Shihunya bukundu*	Shrub	Leaves	4.8	Boil in water
NMA2015/37	Rutaceae	*Zanthoxylum gilletii* (De Wild.) P.G.Waterman	*Shikuma*	Tree	Stem Bark	19.0	Boil in water
NMA2015/38	Solanaceae	*Physalis peruviana* L.	*Mayengo*	Shrub	Leaves	4.8	Inhale steam
NMA2015/39	Solanaceae	*Solanum incanum* L.	*Indalandalu*	Shrub	Root	4.8	Pound, add cold water
NMA2015/40	Verbenaceae	*Lantana trifolia* L.	*Shimenenwa*	Shrub	Leaves	9.5	Boil in water/steam
NMA2015/41	Vitaceae	*Rhoicissus tridentata* (L.f.) Wild and R.B.Drumm.	*Muhoko*	Climber	Root	4.8	Boil in water
NMA2015/42	Xanthorrhoeaceae	*Aloe species*	*Shikakha*	Herb	Leaves	43.0	Boil in water

**Table 2:** Literature review of the plants used for management of malaria among the Luhya community of Kakamega East sub-County.

**Plant name**	**Traditional uses**	***In vitro and in vivo antimalarial activities***	***Antimalarial compounds isolated***
*Justicia betonica* L.	Lower cholesterol, paralysis, earaches, headaches, bruises diarrhea, vomiting, constipation, pain and inflammation and Malaria (Gangabhavani and Ravishankar, 2013),	Ether aerial parts extract had IC_50_ of 13.36 µg/ml (Bbosa et al., 2013)	No reference
*Searsia natalensis* (Bernh.ex C. Krauss)	Malaria (Gathirwa et al., 2011), diarrhea, HIV (Mugisha et al., 2014)	CHCl3 leaf extract had IC_50_ of 1.8 μg/ml (Katuura, et al., 2007b).	No reference
*Carissa edulis* L	Sickle cell anemia, fever, epilepsy, pain (Ya'u et al., 2013), malaria (Orwa et al., 2007)	DCM, stems extract had IC_50_ of 33 μg/ml (Clarkson et al., 2004)	No reference
*Spathodea campanulata* P.Beauv.	Malaria, herpes, fever, diabetes, dysentery, ulcers, HIV (Lim, 2013)	Ethanolic leaf extract had IC_50_ >68 μg/ml (Rangasamy et al., 2008)	Lapachol (Ntie-Kang et al., 2014)
*Markhamia lutea* (Benth.) K.Schum.	Malaria (Lacroix et al., 2009)Anemia, diarrhea, microbial and parasitic infections (Ali et al., 2015)	EtOAc, Leaf extract exhibited 70% parasite suppression (Lacroix et al., 2011)	musambins A-C and musambiosides A-C(Lacroix et al., 2009)
*Warbugia ugandensis* Sprague	Worms, fever, gonorrhea, syphilis (Lacroix et al., 2011) (Were et al., 2010)	DCM, stem bark extract of IC_50_ of 8 μg/ml (Wube et al., 2008) with 69% parasite inhibition (Were et al., 2010)	coloratane sesquiterpenes.(Onguén et al., 2013)
*Vernonia amygdalina* Del.	Febrifuge, vermifuge, laxative, malaria, wounds and as appetizer (Ifeoma and Chukwunonso, 2011)	Ethanolic leaf extract IC_50_ of 9.83 µg/ml (Omoregie et al., 2011)	Vernolide, vernodalin, vernodalol and hydroxyvernolide (Onguén et al., 2013)
		*In-vivo* parasite suppression of between 57.2–72.7% in combination with CQ (Challand and Willcox, 2009)	
*Tithonia diversifolia* (Hemsl.) A. Gray	Diabetes mellitus, sore throat, menstrual pain, malaria, wounds (Owoyele et al., 2004)	ether extract of aerial parts had IC_50_ of 0.75 µg/ ml(Goffin et al., 2002) whereas the methanolic extract had 74% parasitemia suppression (Oyewole et al., 2008)	Tagitinin C (Onguén et al., 2013)
*Acmella caulirhiza* Del.	Toothache, throat and gum infections, dysentery, rheumatism, immune booster and malaria (Grubben, 2004)	DCM extract had IC_50_<10 μg/ml (Owuor et al., 2012)	No reference
*Microglossa pyrifolia* (Lam.) Kuntze	Malaria (Jeruto et al., 2011)Headache, cough, flu, cleansing airway (Moshi et al., 2012)	DCM, Leaf extract had IC_50_ of <15 μg/ml (Muganga et al., 2014)	diterpeness (Kohler et al., 2002)
*Cucumis aculeatus* Cogn	Diarrhea, leprosy, migraines, wounds, gonorrhea (Engels et al., 1991), malaria (Njoroge and Bussmann, 2006)	Aqueous fruit extract had IC_50_ of >30 μg/ml (Gakunju et al., 1995)	No reference
*Crotom macrostachuys* Hochst. ex Del.	Diabetes, dysentery, wounds, malaria, purgative, stomachache (Gelaw et al., 2012)	DCM, leaf extract IC_50_ of 2 μg/ml (Owuor et al., 2012)	No reference
*Harungana madagascariensis* Lam. ex Poir.	Anemia, malaria (Iwalewa et al., 2008), fever, nephrosis, jaundice, gastrointestinal disorders (Okoli et al., 2002)	Ethanolic stem bark extract had IC_50_ of <0.5 μg/ml and showed between 28.6–44.8% Parasite suppression (Iwalewa et al., 2008)	Bazouanthrone, feruginin A, harunganin, harunganol A and B (Ntie-Kang et al., 2014)
*Rotheca myricoides* (Hochst.) Steane and Mabb.	Measles, malaria, asthma, wounds, gonorrhea, rabies and eye disease (Hayelom et al., 2012)	Methanolic leaf extract, IC_50_ of 9.51–10.56 µg/ml and 82% parasite suppression at 600 mg/kg (Deressa et al., 2010)	No reference
*Leucas calostachys* Oliv.	Colds, headache (Okello et al., 2010) malaria (Nyambati et al., 2013)	Aqueous whole plant extract had IC_50_ of 0.8 µg/ml with parasite inhibition of 3.5–5.2% (Nyambati et al., 2013)	No reference
*Ocimun kilimandscharicum* Gürke	Colds, cough, analgesic, sedative, diarrhea, measles (Soni et al., 2012), malaria (Owuor et al., 2012)	DCM, Leaves and twigs had IC_50_ of < 1.5 (Owuor et al., 2012)	No reference
*Fuerstia africana* T.C.E.Fr.	Eye ailments, toothache (Kipkore et al., 2014) malaria (Muganga et al., 2010)	Pet-ether extract of aerial parts had IC_50_ of 1.5 μg/ml (Kigondu et al., 2011)	Ferruginol (Onguén et al., 2013)
		Methanolic leaf extract had IC_50_ <15 μg/ml with > 70% parasite suppression (Muganga et al., 2014)	
*Clerodendrum johnstonii Oliv.*	Abscess, hernia (Quattrocchi, 2012), malaria (Jeruto et al., 2011)	No reference	No reference
*Plectranthus barbatus* Andrews	Gastritis, respiratory disorders, cough, analgesic, hypertension, stomachache, epilepsy (Fernandes et al., 2012) malaria, break fevers (Al-Musayeib et al., 2012)	Methanolic extract had IC_50_ of 6.5 μg/ml, (Al-Musayeib et al., 2012)	No reference
*Ajuga integrifolia* Buch.-Ham	Vermifuge, toothache, hypertension, stomachache fever (Hailu and Engidawork, 2014) malaria (Gitua et al., 2012)	Aqueous leaf extract exhibited 90% parasite suppression (Gitua et al., 2012)	Ajugarin−1(Onguén et al., 2013) ergosterol−5,8-endoperoxide (Ntie-Kang et al., 2014)
*Albizia gummifera* (J.F.Gmel.) C.A.Sm.	Malaria, bacterial infections, skin diseases, stomachache (Kokila et al., 2013)	The alkaloidal fraction had IC_50_ of 0.06 µg/ml while spermine alkaloid exhibited parasite suppression of 43–72% (Rukunga et al., 2007)	spermine alkaloids (Rukunga et al., 2007)
*Senna occidentalis* (L.) Link	Malaria, vermifuge, analgesic, laxative, hepatoprotective, diuretic and febrifuge (Silva et al., 2011)	EtOH root bark extracts had IC_50_<3 μg/ml whereas 200 mg/kg of EtOH and DCM extracts exhibited >60% parasitaemia suppression. (Tona et al., 2001)	Quinones (Kayembe et al., 2010)
*Senna didmobotrya* (Fresen.) H.S.Irwin and Barneby	Intestinal worms, skin diseases, jaundice, veneral diseases, malaria, fever (Nagappan, 2012)	DCM/MeOH. Twigs extract had IC_50_ of 9.5 μg/ml (Clarkson et al., 2004)	No reference
*Erythrina abyssinica* DC.	Abortion, cough, malaria (Lacroix et al., 2011)	EtOAc, Bark extract showed 83% parasite suppression (Lacroix et al., 2011)	5- deoxyabyssinin II and homobutein (Ntie-Kang et al., 2014)
*Trichilia emetica* Vahl	Diabetes, hypertension (Konaté et al., 2014), malaria (Diarra et al., 2015)	DCM/MeOH (1:1), leaves and twigs extract had of IC_50_ of 3.5 μg/ml (Clarkson et al., 2004)	Kurubasch aldehyde (Bero et al., 2009)
*Mellia azedarach* L.	Hepatoprotective, malaria, skin diseases, ulcers, fever, vermifuge, asthma (Qureshi et al., 2016)	DCM, Leaf extract had IC_50_ of 28 μg/ml (Lusakibanza et al., 2010)	No reference
*Cissampelos mucronata* A.Rich	Antisnake venom, veneral diseases, malaria, menstrual disorders, wounds, febrifuge (Nondo et al., 2011)	EtOAc root extract had IC_50_ <3.91 with active compound, curine IC_50_ of 0.24 (Omole, 2012)	curine (Ndiege, 2011)
*Ficus thonningii* Blume	Malaria (Falade et al., 2014), Diabetes, diarrhea, mental illness, gonorrhea, urinary tract infections (Dangarembizi et al., 2013)	Hexane, leaf extract IC_50_ of 10.4 μg/ml (Falade et al., 2014)	No reference
*Flueggea virosa* (Roxb. ex Willd.) Royle	Fever, stomachache, rheumatism, pneumonia, epilepsy, body pains (Magaji et al., 2007) malaria (Al-Rehaily et al., 2015)	MeOH/H_2_O leaves extract had IC_50_ of 7.8 (Willcox et al., 2011a)	securinine and viroallosecurinine (Al-Rehaily et al., 2015)
		Alkaloids: securinine and viroallosecurinine had IC_50_ values of 2.7 and 2.9 μg/ml respectively (Al-Rehaily et al., 2015)	
*Phyllanthus sepialis* Müll. Arg.	Tonic in pregnancy (PROTA, 2008) dental hygiene (Bussmann et al., 2006)	No reference	No reference
*Pittosporum viridiflorum* Sims	Chest complains, purgative, male impotence, asthma, coughs (Dold and Cocks, 2012) malaria (Nyongbela et al., 2013)	DCM whole plant extract had = IC_50_ of 3 μg/ml (Clarkson et al., 2004)	triterpenoid estersaponin, active (Nyongbela et al., 2013)
			pittoviridoside (saponins) (Seo et al., 2002)
*Rumex abyssinicus* Jacq.	Wounds, liver diseases, malaria, gonorrhea (Mulisa et al., 2015)	DCM, root extract had IC_50_ <15 μg/ml, (Muganga et al., 2014)	No reference
*Rumex steudelii* Hochst.ex A. Rich	Antifertility, tonsillitis, wounds, eczema, hemorrhoids, leprosy (Solomon et al., 2010) (Gebrie et al., 2005)	No reference	No reference
*Maesa lanceolata* Forssk	Malaria (Katuura, et al., 2007a)	Chloroform leaf extract IC_50_ of 1.6 µg/ml (Katuura, et al., 2007b)	No reference
		DCM/MeOH (1:1) twigs extract IC_50_ of 5.9 µg/ml (Clarkson et al., 2004)	
*Rubus pinnatus* Wild	Bleeding gums, expectorant, demulcent, diarrhea (Quattrocchi, 2012), malaria (Lacroix et al., 2011)	EtOAc, leaf extract exhibited 20% parasite suppression (Lacroix et al., 2011)	No reference
*Zanthoxylum gilletii* (De Wild.) P.G.Waterman	Stomachache, gonorrhea, back pain, urinogenital infections (Gaya et al., 2013).malaria (Masinde, 2014)	DCM/MeOH (1:1) stem bark extract had IC_50_ of 2.52, 1.48 and 1.43 µg/ml against W2, D6 and 3D7 strains. (Masinde, 2014)	Nitidine (Muganga et al., 2014) Seasamine
			8-acetonyldihydrochelerythrine (Masinde, 2014)
*Clausena anisata* (Willd.) Hook.f. ex Benth.	vermifuge, febrifuge, measles, hypertension, malaria, analgesic, rheumatism (Okokon et al., 2012)	DCM, root extract had IC_50_ of 10 μg/ml (Clarkson et al., 2004)	No reference
*Physalis peruviana* L.	malaria, rheumatism, hepatitis, dermatitis, diuretic (Ramadan et al., 2015)	No reference	No reference
*Solanum incanum* L.	Pneumonia, liver pain, headache, toothache, stomachache, sore throat (PROTA, 2008), malaria (Nguta et al., 2010)	CHCl_3_/MeOH, leaf extract showed 31% parasite suppression (Murithi et al., 2014)	No reference
*Lantana trifolia* L.	Common cold, asthma, epilepsy, madness, childhood cerebral malaria, sickle cell anemia.(Nalubega et al., 2013)	Pet-ether aerial parts extract had IC_50_ of 13.2 μg/ml (Katuura et al., 2007b)	No reference
*Rhoicissus tridentata* (L.f.) Wild and R.B.Drumm.	Dysmenorrhea, uterotonic, indigestion, pregnancy and childbirth. (Mukundi et al., 2015), malaria (Gakunju et al., 1995)	Aqueous root extract had IC_50_ > 40 μg/ml (Gakunju et al., 1995)	No reference
*Aloe species*	Colds, malaria, stomachache, anemia (PROTA, 2008)	Ether leaf extract *of A.dawei*, IC_50_ of 7.9 μg/ml (Bbosa et al., 2013)	No reference

CHCl_3_=Chloroform, DCM=Dichloromethane, EtOAc=Ethyl Acetate, MeOH=methanol, Pet-ether=Petroleum ether
