# Supplementary material for: Medicinal plants used for management of malaria among the Luhya community of Kakamega East sub-County, Kenya
Source: J Ethnopharmacol. 2016 Dec 24;194:98–107. doi: 10.1016/j.jep.2016.08.050 (PMC5176009; doi:10.1016/j.jep.2016.08.050)
Supplement: Supplementary file 1 — Supplementary material [file mmc1.docx]

**Data Collecting Tool**

1. **Eligibility Checklist**
2. Has lived in Kakamega County for one year? Yes/No
3. Do you agree to take part in this study? Yes/No
4. Have you used plants in management of malaria? Yes/No
5. **Demographic information**

Sex..............................................................................................

Age...................................................................................................

Religion…………………………………………………………….

Village/Area of residence..................................................................

Level of Education (Tick)

| No formal education |  |
| --- | --- |
| Primary |  |
| Secondary |  |
| Tertiary |  |
| Technical/Vocational |  |

Type of practitioner

| Herbalist practitioner |  |
| --- | --- |
| Care giver in household |  |

1. **Knowledge of malaria**
2. What is malaria? *(Ask about the vernacular name and the causes of malaria*).
3. Have you managed someone suffering from malaria using herbs? Yes/No
4. If yes, how did the patients present? *( Probe about the symptoms of malaria known to the practitioner)*
5. **Plants used in management of malaria**
6. Mention the plants you have used in malaria management
7. --------------------------------------------
8. ------------------------------------------
9. ----------------------------------------------------
10. ----------------------------------------------------
11. ------------------------------------------------------
12. -----------------------------------------------------
13. --------------------------------------------------------
14. -------------------------------------------------------------
15. ------------------------------------------------------------------
16. ------------------------------------------------------

***The form below will be filled for each plant mentioned above.***

**Data about medicinal plant and its use:**

Plant (Local name).............................................................................

Habit (Tree/ Herb/ Shrub/Climber/…...)

Identification features…………………………………….

…………………………………..

……………………………………..

………………………………………….

Plant part used...................................................................................

Cultivated/ Wild................................................................................

How is the availability of the plant in the plant in this area?

| Easily available |  |
| --- | --- |
| Sometimes |  |
| Rarely |  |
| Not found |  |

Method of plant preparation

|  |
| --- |

Mode of administration.......................................................................

| By mouth |  |
| --- | --- |
| inhalation |  |
| Taking bath |  |
| Application on the skin |  |
| Others (specify) |  |

Dosage (amount) Adults...........................................................................................

Children…………………………………………………………….

Frequency of administration …………………………………………………………………….

Duration of usage ……………………………………………………………………

Can this plant be used by pregnant women?

When was the last time you used this plant?

| Less than 30 days ago |  |
| --- | --- |
| More than a month |  |
| Last year |  |

Any other plant(s) used together with the plant

If mixed, how do you mix the various components?

What is the role of each of the components?

Other uses (if any)…………………………......……...........................

Side effects associated with use of the plant.

Any special considerations necessary for the plant

(E.g time of harvesting, geographical location, maturity, season to harvest).

Is the respondent willing to show the plants they use? If yes, conduct a field visit and collect samples.

**Remarks:**

Plant identified as ………………………………………… (Botanical name and family)

Signature of Researcher
